# Gut Microbiome and Disorders of the Gastrointestinal Tract

**DOI:** 10.3390/microorganisms12030576

**Published:** 2024-03-13

**Authors:** Alexander Hammerhøj, John Mark Gubatan, Ole Haagen Nielsen

**Affiliations:** 1 Department of Gastroenterology, Herlev Hospital, University of Copenhagen, DK-2730 Herlev, Denmark; alexander.hammerhoej@regionh.dk; 2 Department of Gastroenterology and Hepatology, Stanford University Medical Center, Stanford, CA 95305, USA; jgubatan@stanford.edu

The protective intestinal epithelial barrier is constantly exposed to more than 100 trillion commensal microorganisms (bacteria, archaea, viruses, and fungi), i.e., the microbiome, making the human gastrointestinal tract a highly active and dynamic ecosystem [1]. Recent developments in gut microbiota research have unveiled its profound impact on human health via multiple key functions through a dynamic and complex network of interactions with the host [1]. Observational studies and advancements in genomic sequencing have enabled a deeper understanding of the gut microbiome’s complexity and diversity and its importance for maintaining gut homeostasis and digestive health, including the digestion of food, the production of vitamins, the absorption of micronutrients, immune homeostasis, the maintenance of the gut barrier function, and protection against pathogens [1]. Moreover, dysbiosis may lead to a proinflammatory environment that can cause bacterial translocation, systemic immune activation, tissue damage, and carcinogenesis [2]. In order to precisely characterize the intestinal microbiome in humans, it is important to use a wide range of molecular techniques to explore the response to antimicrobial therapy, including links between long-lasting symptom resolution and the microbiome. Such efforts may pave the path for novel diagnostic and therapeutic approaches that will ultimately allow us to define the role of microbes behind the pathology of a number of disorders which may potentially individualize treatments and improve patient outcomes.

One noteworthy development revolves around the concept of microbiome-based diagnostics. Researchers are exploring the use of specific microbial signatures as biomarkers for various health conditions, offering non-invasive and potentially more accurate means of disease detection and monitoring [3]. By sequencing rRNA, microbial profiles aim to provide novel valuable diagnostic tools. Likewise, microbial analysis also holds potential for the prediction of conditions at an early point.

An intricate balance between tolerance to commensal microorganisms and protection against enteric pathogens is required to maintain intestinal homeostasis. Perturbations in the composition and function of the gut microbiota are recognized to play a critical role in the pathogenesis of various disorders of the gastrointestinal tract, and in dysbiotic conditions, gut microbes may contribute to the development of a wide spectrum of diseases, including metabolic syndrome, intestinal microinflammation, non-alcoholic fatty liver disease, and inflammatory bowel disease [4]. Moreover, the interaction between gut microbiota and the host’s immune system is pivotal, as anti-commensal IgG is increased in ulcerative colitis, where it may even instigate disease exacerbation [5], whereas an increase in both IgG- and IgA-bound commensal bacteria is observed in the gut microbiota in active Crohn’s disease [6].

As the understanding of beneficial microbiota composition strengthens, so does the prospect of employing helpful bacteria in the treatment of intestinal disorders. Personalized therapeutic microbiota interventions, such as prebiotics, probiotics, and fecal microbiota transplantation, hold promise for treating various diseases. The unique microbial fingerprint of each individual can lead to tailored approaches for modulating gut health.

Nonetheless, novel insights into the role of specific microbes in diverse digestive diseases hold great translational potential for patients with a wide range of gastrointestinal disorders [7]. This Special Issue of Microorganisms, which focused on the current landscape, explores interactions between the gut microbiome and gastrointestinal diseases in the context of disease pathogenesis and emerging microbial-based biomarkers and therapies. Moreover, new research data that addressed these topics were published and shed light on the critical contribution of the gut microbiota in the pathogenesis of various GI disorders and its potential capacity for developing efficient biomarkers and therapeutic strategies. Finally, the impact of climate change on the development of diseases in the GI tract is increasingly recognized [8]. A major number of infectious diseases may be aggravated by climate change, and by 2030, a 10% spike in diarrheal illness is anticipated [7,9], and the occurrence of IBD also seems to be affected by the climate [10].

As the field of microbiota research continues to evolve, several key areas warrant focused attention for future investigations. Thus, a deeper understanding of the functional role of specific microbial species and their metabolites is crucial. While advancements in metagenomic sequencing have enabled the identification of various microorganisms within the microbiota, unraveling their specific contributions to health and disease still remains a complex task. As interventions like fecal microbiota transplants and microbial modulators gain popularity, understanding their potential risks and unintended consequences is essential to ensure the safe application of such therapies in the future.
